# Pelvic Pain Symptoms and Inflammation Among Adolescents and Adults with and Without Endometriosis

**DOI:** 10.3390/ijms26115377

**Published:** 2025-06-04

**Authors:** Amy L. Shafrir, Ashley Laliberte, Britani Wallace, Allison F. Vitonis, Christine B. Sieberg, Marzieh Ghiasi, Larry I. Magpantay, Marta Epeldegui, Andrew Schrepf, Sawsan As-Sanie, Kathryn L. Terry, Stacey A. Missmer

**Affiliations:** 1 Department of Health Sciences and Nutrition, School of Nursing and Health Sciences, Merrimack College, North Andover, MA 01845, USA; 2 Division of Adolescent and Young Adult Medicine, Department of Pediatrics, Boston Children’s Hospital and Harvard Medical School, Boston, MA 02115, USA; csieberg@mgh.harvard.edu (C.B.S.); missmer@med.umich.edu (S.A.M.); 3 Boston Center for Endometriosis, Boston Children’s Hospital and Brigham and Women’s Hospital, Boston, MA 02115, USA; alaliberte3@bwh.harvard.edu (A.L.); avitonis@bwh.harvard.edu (A.F.V.); kterry@bwh.harvard.edu (K.L.T.); 4 Department of Obstetrics, Gynecology, and Reproductive Biology, Brigham and Women’s Hospital and Harvard Medical School, Boston, MA 02115, USA; 5 Center for Health Outcomes and Interdisciplinary Research, Department of Psychiatry, Massachusetts General Hospital, Boston, MA 02114, USA; 6 Department of Psychiatry, Harvard Medical School, Boston, MA 02115, USA; 7 Department of Internal Medicine, Cleveland Clinic, Cleveland, OH 44195, USA; ghiasim@ccf.org; 8 UCLA AIDS Institute and David Geffen School of Medicine, University of California, Los Angeles, CA 90095, USA; lmagpant@ucla.edu (L.I.M.); mepeldegui@mednet.ucla.edu (M.E.); 9 Department of Obstetrics and Gynecology, David Geffen School of Medicine, University of California, Los Angeles, CA 90095, USA; 10 Jonsson Comprehensive Cancer Center, University of California, Los Angeles, CA 90024, USA; 11 Department of Anesthesiology, University of Michigan, Ann Arbor, MI 48109, USA; aschrepf@med.umich.edu; 12 Department of Obstetrics and Gynecology, University of Michigan, Ann Arbor, MI 48109, USA; sassanie@med.umich.edu; 13 Department of Epidemiology, Harvard T.H. Chan School of Public Health, Boston, MA 02115, USA

**Keywords:** pelvic pain, endometriosis, inflammation, dysmenorrhea, dyspareunia, bowel pain, cytokines, chemokines

## Abstract

We evaluated inflammatory markers among 389 surgically confirmed endometriosis cases and 505 controls from the Women’s Health Study: From Adolescence to Adulthood (A2A) cohort. Participants reported dysmenorrhea, acyclic pelvic pain, dyspareunia, and pain with bowel movements. Using multiplex assays, we measured their levels of plasma interleukin (IL)-1β, -6, -8, -10, and -16, tumor necrosis factor (TNF)-α, monocyte chemotactic protein (MCP)-1 and -4, thymus and activation-regulated chemokine (TARC), and interferon gamma-induced protein (IP)-10. For each symptom, we computed biomarker-level geometric means (GMs) with 95% confidence intervals (95% CI) using multivariate linear regression among the endometriosis cases and controls, with interactions with case/control status tested using Wald statistics. Among the controls, those with dyspareunia had lower levels of IL-8 (GM_present_ = 4.64 [95% CI = 4.41–4.89] pg/mL vs. GM_absent_ = 4.99 [95% CI = 4.82–5.17] pg/mL; *p* = 0.02), and the IL-8 levels were lower for controls reporting pain with bowel movements (GM_present_ = 4.66 [95% CI = 4.43–4.89] vs. GM_absent_ = 4.96 [95% CI = 4.82–5.11] pg/mL, *p* = 0.03). No significant associations between pelvic pain symptoms and inflammatory markers were observed among the endometriosis cases; however, the relationship between inflammatory marker levels and pain experience varied by analgesic use at blood draw. Dyspareunia and pain with bowel movements were associated with inflammatory markers among the controls, while the associations between pelvic pain symptoms and inflammatory markers among the endometriosis cases differed by analgesic use.

## 1. Introduction

Pelvic pain affects many reproductive-aged individuals, with 35–90% experiencing dysmenorrhea [1] and 15–20% experiencing chronic acyclic pelvic pain [2,3], resulting in a reduced quality of life, decreased work productivity, and substantial healthcare costs [1,2,3]. Recent evidence suggests that women with moderate and/or severe pelvic pain may be at an increased risk of having a co-existing inflammatory condition, including migraines and allergies, suggesting that systemic inflammation may play a role in pelvic pain symptoms [4]. While there are various causes for pelvic pain among reproductive-aged individuals, approximately 28% of those with chronic pelvic pain have endometriosis [5,6,7], and upwards of two-thirds of adolescents with treatment-resistant chronic pelvic pain or life-impacting dysmenorrhea have endometriosis [8,9]. Endometriosis, characterized by endometrial-like tissue thriving outside of the uterus, affects approximately 10% of reproductive-aged adolescents and adults and is an often chronic, inflammatory disease with heterogeneity in terms of its symptom presentation, lesion characteristics, and response to standard treatments [10,11].

Although the etiology and pathogenesis of endometriosis are not fully understood, inflammation and immune dysregulation are key components [12,13,14,15,16]. The peritoneal fluid of women with endometriosis contains greater numbers of immune cells, potentially enhancing the ectopic endometrial cells’ survival and proliferation through the secretion of various local products (e.g., growth factors and cytokines) [12,17]. Previous research has noted increased levels of macrophages and neutrophils and decreased cytotoxicity of natural killer cells in addition to shifts in macrophage activation in the peritoneum of women with endometriosis [11,18,19]. The presence of endometriotic lesions in the peritoneal cavity leads to the recruitment of inflammatory molecules that can irritate nerve endings, potentially inducing endometriosis-associated pain [20,21]. Additionally, increased systemic inflammatory activity has been associated with greater central sensitization of pain in other forms of pelvic pain, including urologic pelvic pain [22,23]. Furthering our understanding of the mechanisms underlying pelvic pain symptoms has the potential to lead to advances in targeted and more precise pain symptom management.

Previous studies of the relationship between inflammatory molecules within peritoneal fluid and endometriosis-associated pain have yielded inconsistent results, with a limited overlap in the cytokines and chemokines investigated [24]. While one study noted a positive correlation between the level of tumor necrosis factor (TNF)-α in peritoneal fluid and dysmenorrhea symptoms among women with endometriosis [25], other studies have found no association [26,27]. Interleukin (IL)-1β has consistently been reported at higher levels in the peritoneal fluid of women with endometriosis and pelvic pain compared to that of control women [28,29]. While evaluating cytokine and chemokine levels in peritoneal fluid provides a localized assessment of inflammation and endometriosis-associated pain symptoms, the control groups in these studies included women who underwent surgery for other benign pelvic pathologies or parous women undergoing tubal ligation. If the presence of other pelvic pathologies or multiparity are also related to inflammatory changes, this may have led to erroneous results [10].

Only a few studies have assessed inflammatory markers in blood samples and endometriosis-associated pain symptoms, and two of these were restricted to women with endometriomas [30,31,32,33,34]. One case–control study (*n* = 112 women with endometriosis and 107 controls) noted that decreased levels of IL-19 and -22 were associated with deep dyspareunia and acyclic pelvic pain among women with endometriomas [30]. In one case series of women with endometriomas (*n* = 51), no correlation was observed between IL-8 and severe pelvic pain, which was defined as the presence of treatment-resistant dysmenorrhea and/or dyspareunia and/or chronic pelvic pain [31]. Conversely, a study of women with infertility (*n* = 44 women with endometriosis and 43 controls) noted higher IL-8 and IL-12p70 levels among those with pelvic pain compared to those without [32]. Additionally, IL-16 levels have been observed to be higher among women with endometriosis and severe chronic pelvic pain (*n* = 65) compared to those with endometriosis and mild pain (*n* = 65) [33]. Finally, IL-6 levels were found to be highest in patients with endometriosis and chronic pelvic pain compared to those with endometriosis without chronic pelvic pain and to control women undergoing laparoscopic tubal ligation (*n* = 85 cases and 53 controls) [34]. However, none of these studies controlled for potential confounders and only included control participants who had undergone laparoscopic surgery (thus all presenting with gynecologic pathology-associated symptoms). Additionally, these studies included older populations with mean ages of >30 years, potentially missing important inflammatory processes during the adolescent and young adult period.

In order to investigate the associations between pelvic pain and inflammation and the potential effect of endometriosis, we sought to understand whether levels of circulating cytokines and chemokines and pelvic pain symptoms differed between and within adolescents and adults with and without endometriosis. Notably, the participants with endometriosis in our analysis primarily comprised those with only superficial peritoneal lesions and revised American Society for Reproductive Medicine (rASRM) stage I/II disease, the most common endometriosis subphenotype [35], while those without endometriosis intentionally primarily comprised individuals recruited from the general population to minimize the oversampling of those with non-endometriosis gynecologic pathologies that may also influence the inflammatory milieu. We investigated the association between the presence, severity, and frequency of dysmenorrhea, acyclic pelvic pain, and dyspareunia, as well as the presence of pain with bowel movements, and circulating cytokine and chemokine (C-reactive protein (CRP), IL-1β, IL-6, IL-8, IL-10, TNF-α, monocyte chemotactic protein (MCP)-1, MCP-4, thymus and activation-regulated chemokine (TARC), IL-16, interferon gamma-induced protein (IP)-10) levels among adolescents and adults with and without endometriosis. These inflammatory markers were selected to represent pro-inflammatory, regulatory, and chemotactic aspects of the inflammatory response.

## 2. Results

We included 389 participants with surgically diagnosed endometriosis and 505 control participants without a surgical diagnosis of endometriosis. Compared to controls, the endometriosis cases were more likely to be younger (median age: 17 vs. 24 years) and White (91% vs. 72%) and report hormonal medication use within the 30 days prior to the blood draw (88% vs. 56%; Table 1). Fewer endometriosis cases reported having periods in the last 3 months (65%) compared to controls (85%). Among the endometriosis cases, 95% had rASRM stage I/II disease and only superficial peritoneal lesions based on their surgery closest to the blood draw. The median age at first endometriosis symptoms was 13 years old (interquartile range (IQR): 12 to 15 years), and the median time from the first symptoms to surgical diagnosis was 2 years (IQR: 1, 5).

Adjusting for participants’ age, body mass index (BMI), hormone use within the 30 days prior to the blood draw, and pain medication use within the 48 h prior to the blood draw, higher inflammatory marker levels were noted for endometriosis cases compared to controls for each of the inflammatory markers, with the exception of IL-6, IL-10, and TARC, for which the levels were higher among the controls compared to the endometriosis cases (Table 2). The differences in inflammatory marker levels were similar between the cases and controls with additional adjustments for dysmenorrhea severity and presence of acyclic pelvic pain (Tables S1 and S2).

**Figure 1:**
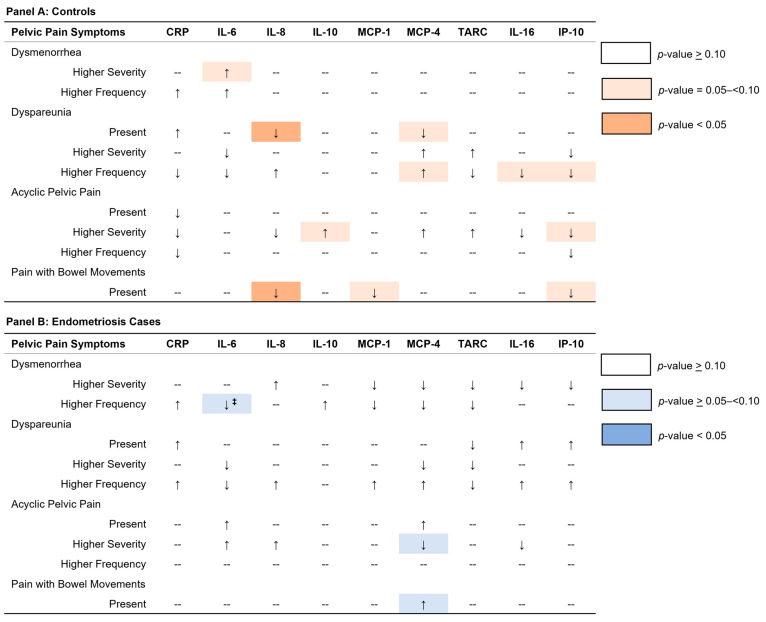
Overall direction and magnitude of effect for associations between pelvic pain symptoms and plasma cytokine and chemokine levels among A2A participants with and without endometriosis are presented in Panels (**A**,**B**). Arrows show direction of association for changes in geometric mean biomarker levels of 10% or greater or where *p*-value for association is <0.10. Panel (**A**) displays direction of association (arrows) and strength of association (shaded boxes) for controls. Panel (**B**) shows direction of association (arrows) and strength of association (shaded boxes) for endometriosis cases. Double dagger indicates significant heterogeneity between endometriosis cases and controls for association between pelvic pain symptoms and inflammation markers.

Summary results for the associations between pelvic pain symptoms and the inflammation biomarkers are presented in Figure 1 and show the direction of association and magnitude of the *p*-value. Pelvic pain symptom-specific details in relation to the inflammation markers are presented below.

### 2.1. Dysmenorrhea

**Figure 2:**
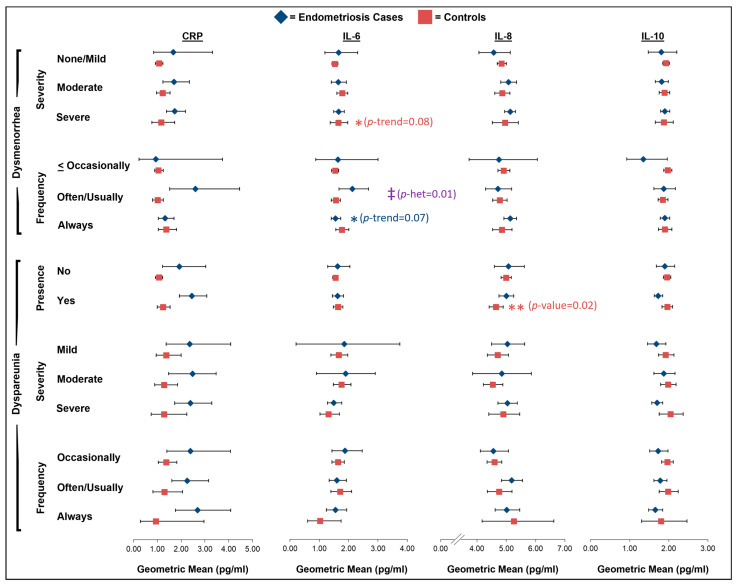
Geometric mean levels of CRP, IL-6, IL-8, and IL-10 in relation to dysmenorrhea severity and frequency and presence, severity, and frequency of dyspareunia among endometriosis cases (blue diamonds) and controls (red squares) in A2A cohort. All estimates were adjusted for age at blood draw (continuous), body mass index at blood draw (under-/normal weight, overweight, obese), pain medication use within 48 h of blood draw (yes, no, missing), and hormone use within 30 days of blood draw (yes, no). Asterisks (*) denote borderline and significant *p*-values for trend (frequency and severity) and *p*-values (presence) (no asterisk: *p*-value > 0.10; one asterisk: *p*-value = 0.05–<0.10; two asterisks: *p*-value < 0.05). Double dagger symbols (‡) denote significant heterogeneity (*p*-het < 0.05) between endometriosis cases and controls. Full results, including sample size numbers, can be found in Table S3.

**Figure 3:**
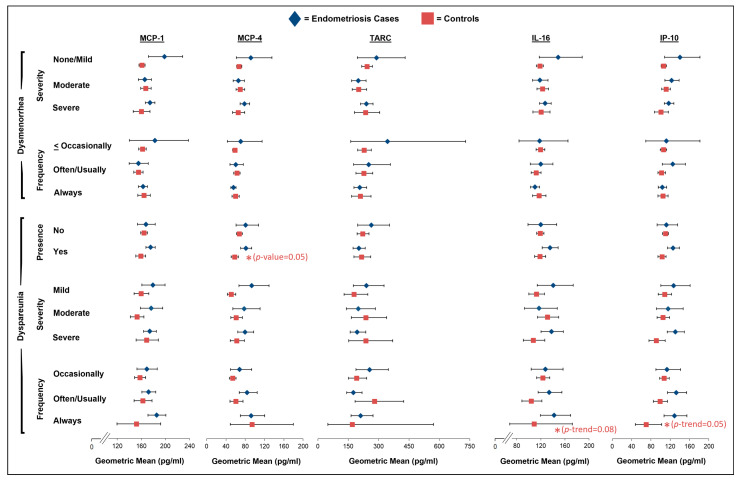
Geometric mean levels of MCP-1, MCP-4, TARC, IL-16, and IP-10 in relation to dysmenorrhea severity and frequency and presence, severity, and frequency of dyspareunia among endometriosis cases (blue diamonds) and controls (red squares) in A2A cohort. All estimates were adjusted for age at blood draw (continuous), body mass index at blood draw (normal weight, overweight, obese), pain medication use within 48 h of blood draw (yes, no, missing), and hormone use within 30 days of blood draw (yes, no). Asterisks (*) denote borderline and significant *p*-values for trend (frequency and severity) and *p*-values (presence) (no asterisk: *p*-value > 0.10; one asterisk: *p*-value = 0.05–<0.10). Full results, including sample size numbers, can be found in Table S3.

Regarding dysmenorrhea severity, there was a non-statistically significant suggestion that controls with severe dysmenorrhea had higher levels of IL-6 (geometric mean (GM): 1.64; 95% CI: 1.37–1.97) compared to the controls with no/mild dysmenorrhea (GM: 1.53; 95% CI: 1.43–1.63; *p*-trend = 0.08), but no association was noted among the endometriosis cases (*p*-trend = 0.95; Figure 2; Table S3). We observed a non-significant inverse association between dysmenorrhea frequency and IL-6 levels among the cases (GM for often/usually of 2.12 with 95% CI of 1.67–2.68 vs. GM for always of 1.55 with 95% CI of 1.40–1.73; *p*-trend = 0.07) with a significant interaction with case–control status (*p*-interaction = 0.01), as no association was observed among the controls (*p*-trend = 0.11; Figure 2; Table S3). There was no significant effect modification by analgesic use; however, the positive association between dysmenorrhea severity and IL-6 levels among the controls appeared to be driven by the controls who reported using analgesics (*p*-trend = 0.04; Table S4). Additionally, no other linear associations were observed in the main analyses between any of the cytokines or chemokines and dysmenorrhea severity or frequency for the endometriosis cases or controls (Figure 2 and Figure 3; Table S3).

In the secondary analyses, there was a non-statistically significant suggestion that dysmenorrhea severity increased with increasing TNF-α levels among the controls (GM for none/mild of 6.94 with 95% CI of 6.70–7.19 vs. GM for severe of 7.18 with 95% CI of 6.41–8.05; *p*-trend = 0.07), with a significant interaction between dysmenorrhea severity and TNF-α levels between the cases and controls (*p*-int = 0.04) when the TNF-α levels were measured using the Ella platform (Table S5). This association appeared to be stronger among the controls who did not use analgesics within the 48 h prior to the blood draw (Table S4). Conversely, we observed no association between dysmenorrhea severity and TNF-α levels (*p*-trend = 0.11) among the controls when the TNF-α levels were measured using the Luminex platform (Table S5). Regarding the categories for the IL-1β levels, the endometriosis cases who experienced dysmenorrhea often/usually were 79% less likely to be in the category with the highest IL-1β levels (between the median and highest values) compared to the endometriosis cases who always experienced dysmenorrhea (odds ratio (OR): 0.21; 95% CI: 0.06–0.74; Table S6).

In the sensitivity analyses excluding those who had taken steroid medications within the 48 h prior to the blood draw, the association between dysmenorrhea severity and IL-6 among the controls was attenuated, while the association between dysmenorrhea severity and TNF-α as well as that between dysmenorrhea frequency and IL-1β and IL-6 (Tables S7 and S8) changed negligibly. Excluding participants with extrapolated biomarker levels, we observed similar associations between dysmenorrhea severity and IL-6 (Table S9).

### 2.2. Dyspareunia

The control participants with dyspareunia had significantly lower levels of IL-8 and MCP-4 compared to the control participants without dyspareunia in the past 12 months (IL-8: GM for yes of 4.64 with 95% CI of 4.41–4.89 vs. GM for no of 4.99 with 95% CI of 4.82–5.17, *p* = 0.02; MCP-4: GM for yes of 57.7 with 95% CI of 50.7–65.6 vs. GM for no of 67.7 with 95% CI of 61.9–73.9, *p* = 0.05; Figure 2 and Figure 3; Table S3). Although there was no significant effect modification by analgesic pain medication use, the association between the presence of dyspareunia and levels of IL-8 and MCP-4 appeared to be stronger among the controls who had not used analgesics at the time of the blood draw (Table S4). Additionally, among the controls, there was a significant inverse association between dyspareunia frequency and IP-10 levels (GM for occasionally of 107.4 with 95% CI of 97.9–117.8 vs. GM for always of 70.1 with 95% CI of 47.9–102.6; *p*-trend = 0.05) and a non-significant inverse association with IL-16 levels (GM for occasionally of 123.5 with 95% CI of 113.0–134.9 vs. GM for always of 108.8 with 95% CI of 68.8–172.3; *p*-trend = 0.08; Figure 3; Table S3). No other linear associations were observed in the main analyses between any of the cytokines or chemokines and the presence, severity, or frequency of dyspareunia among the endometriosis cases or controls (Figure 2 and Figure 3, Table S3).

In the secondary analyses, we observed a significant decrease in TNF-α with increasing dyspareunia frequency using the Ella platform (*p* = 0.008) and a non-significant association with a similar direction of association using the Luminex assay (*p* = 0.25; Table S5). For IL-1β, we noted higher levels of IL-1β among the endometriosis cases with moderate dyspareunia as opposed to severe dyspareunia (OR: 5.40; 95% CI: 1.35–21.6; Table S6).

In the sensitivity analyses, the inverse association between IL-8 and the presence of dyspareunia among the controls became stronger when participants using steroid medications at the blood draw were removed (*p* = 0.009; Table S7). The associations with MCP-4, TNF-α, and IL-1β were similar while the associations with IP-10 and IL-16 were attenuated but in the same direction when removing participants who used steroid medications (Tables S7 and S8). When participants with extrapolated values for their IL-8 levels were removed, we observed similar results to those from the main analyses (Table S9).

### 2.3. Acyclic Pelvic Pain

**Figure 4:**
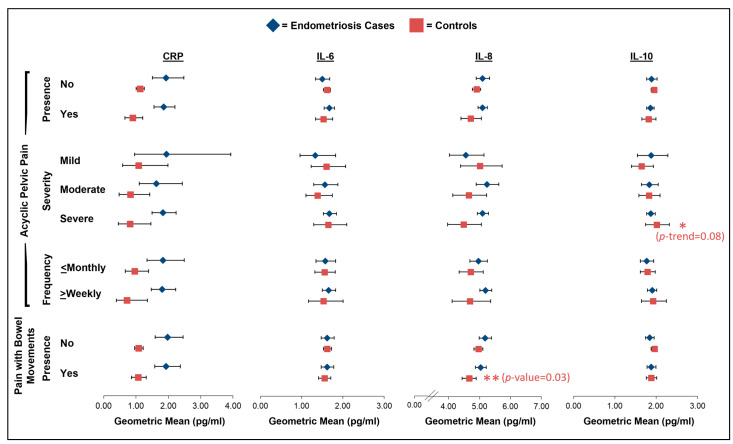
Geometric mean levels of CRP, IL-6, IL-8, and IL-10 in relation to presence, severity, and frequency of acyclic pelvic pain and presence of pain with bowel movements among endometriosis cases (blue diamonds) and controls (red squares) in A2A cohort. All estimates were adjusted for age at blood draw (continuous), body mass index at blood draw (normal weight, overweight, obese), pain medication use within 48 h of blood draw (yes, no, missing), and hormone use within 30 days of blood draw (yes, no). Asterisks (*) denote borderline and significant *p*-values for trend (frequency and severity) and *p*-values (presence) (no asterisk: *p*-value < 0.10; one asterisk: *p*-value = 0.05–<0.10; two asterisks: *p*-value < 0.05). Full results, including sample size numbers, can be found in Table S3.

**Figure 5:**
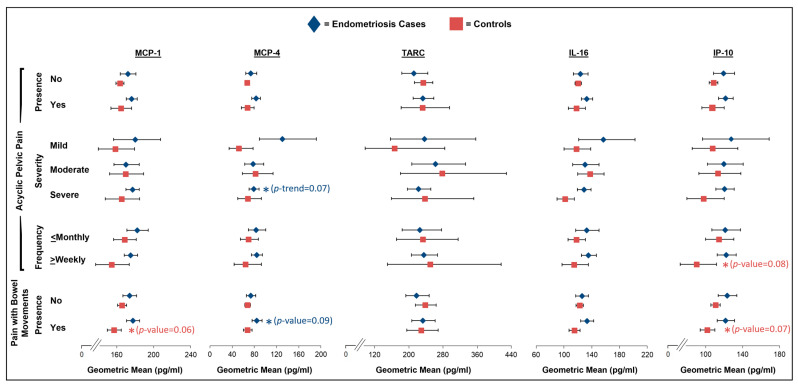
Geometric mean levels of MCP-1, MCP-4, TARC, IL-16, and IP-10 in relation to presence, severity, and frequency of acyclic pelvic pain and presence of pain with bowel movements among endometriosis cases (blue diamonds) and controls (red squares) in A2A cohort. All estimates were adjusted for age at blood draw (continuous), body mass index at blood draw (normal weight, overweight, obese), pain medication use within 48 h of blood draw (yes, no, missing), and hormone use within 30 days of blood draw (yes, no). Asterisks (*) denote borderline and significant *p*-values for trend (frequency and severity) and *p*-values (presence) (no asterisk: *p*-value < 0.10; one asterisk: *p*-value = 0.05–<0.10). Full results, including sample size numbers, can be found in Table S3.

Regarding acyclic pelvic pain severity, we observed a non-statistically significant positive association between greater acyclic pelvic pain severity and increasing IL-10 levels among the controls (GM for mild of 1.64 with 95% CI of 1.40–1.93 vs. GM for severe of 2.01 with 95% CI of 1.74–2.32; *p*-trend = 0.08; Figure 4; Table S3). Additionally, there was an indication that MCP-4 levels decreased with increasing severity among the endometriosis cases (GM for mild of 130.8 with 95% CI of 89.4–191.5 vs. GM for severe of 78.9 with 95% CI of 70.6–88.3; *p*-trend = 0.07; Figure 5; Table S3). We additionally observed a borderline association between acyclic pelvic pain frequency and IP-10 among the controls with more frequent acyclic pelvic pain, associated with lower levels of IP-10 (GM for monthly or less of 114.3 with 95% CI of 100.0–130.6 vs. GM for weekly/daily of 90.1 with 95% CI of 72.6–111.8; *p* = 0.08; Figure 5; Table S3). While no association between the presence of acyclic pelvic pain and IL-8 levels was observed among the endometriosis cases (Figure 4; Table S3), we did note a borderline significant interaction with analgesic use for the association between the presence of acyclic pelvic pain and IL-8 among the cases (*p*-interaction = 0.06; Table S10). The endometriosis cases who reported using analgesics prior to the blood draw and had acyclic pelvic pain had lower levels of IL-8 compared to those without acyclic pelvic pain (*p* = 0.05), while no association between acyclic pelvic pain and IL-8 was observed for the endometriosis cases who were not taking analgesics prior to the blood draw.

In the secondary analyses, there were no significant associations between acyclic pelvic pain and TNF-α or IL-β (Tables S5 and S6). When the analysis was restricted to participants who did not use steroid medications within 48 h of the blood draw, the associations with IL-10, MCP-4, and IP-10 were all in the same direction but attenuated. Additionally, there was a significant positive association between acyclic pelvic pain severity and MCP-4 levels among the controls (*p*-trend = 0.04; Table S7). Removing participants with extrapolated IL-10 levels attenuated the association; however, the trend was still in the same direction, and there was a significant association between the presence of acyclic pelvic pain and IL-10 levels, with lower IL-10 levels among the controls with acyclic pelvic pain compared to the controls without (*p* = 0.05; Table S9).

### 2.4. Pain with Bowel Movements

In the main analyses of pain with bowel movements, the controls reporting pain with bowel movements had significantly lower levels of IL-8 (GM: 4.66; 95% CI: 4.43–4.89) compared to the controls without pain with bowel movements (GM: 4.96; 95% CI: 4.82–5.11; *p* = 0.03; Figure 4, Table S3), for which there was no evidence of effect modification by analgesic pain medication use (Table S4). We also noted that the levels of MCP-1 and IP-10 were lower among the controls with pain with bowel movements compared to the controls without (*p* = 0.06 and *p* = 0.07, respectively; Figure 5; Table S3). Although there was no significant effect modification by analgesic pain medication use for the association between pain with bowel movements and MCP-1 and IP-10 levels among the controls, the associations appeared to be stronger among the controls who did not use analgesics prior to the blood draw (Table S4). Finally, we observed non-statistically significant higher levels of MCP-4 among the endometriosis cases with pain with bowel movements compared to those without (*p* = 0.09; Figure 5; Table S3), with significant effect modification by analgesic pain medication use (*p*-interaction = 0.02; Table S10). The endometriosis cases who were not using analgesics and had pain with bowel movements had higher levels of MCP-4 compared to those who did not have pain with bowel movements (*p* = 0.04); however, an association in the opposite direction was observed for the endometriosis cases who were using analgesics (*p* = 0.13).

In the secondary analyses, the endometriosis cases with pain with bowel movements had lower levels of TNF-α (GM: 7.18; 95% CI: 6.66–7.73) compared to those without pain with bowel movements (GM: 8.06; 95% CI: 7.41–8.77; *p* = 0.05; Table S5) when measured using Ella. Similar results were noted using the Luminex assay, although the association was not significant (*p* = 0.64). Additionally, there was an indication of effect modification by analgesic pain medication use (*p*-het = 0.07), with a significant inverse association between pain with bowel movements and TNF-α among the endometriosis cases not using analgesics (*p* = 0.009) but a non-significant positive association among the cases using analgesics at the blood draw (*p* = 0.33; Table S10). The endometriosis cases with pain with bowel movements were less likely to have IL-1β levels that were above the lower limit of detection or higher compared to those without pain with bowel movements (Table S6).

In the sensitivity analyses, the results were similar for MCP-1, IP-10, TNF-α, and IL-1β when participants taking steroid medications were removed (Tables S7 and S8). Additionally, the results for IL-8 were attenuated but in the same direction when participants taking steroid medications were removed (Table S7). The results for IL-8 were similar when participants with extrapolated values were removed (Table S9).

## 3. Discussion

In this cross-sectional analysis, we evaluated the associations between the circulating cytokine and chemokine levels and the presence, severity, and frequency of dysmenorrhea, acyclic pelvic pain, dyspareunia, and pain with bowel movements among adolescents and adults with and without endometriosis with adjustment for important confounding factors, including hormonal and pain medication use. While none of the main associations were statistically significant among the endometriosis cases, there was an indication of effect modification by analgesic use prior to the blood draw. Among the endometriosis cases using analgesics, there was an indication that those who had acyclic pelvic pain had lower levels of IL-8 compared to the cases without acyclic pelvic pain. There was also an indication of effect modification by analgesic use for pain with bowel movements and the level of MCP-4, as the endometriosis cases who were not taking analgesics and had pain with bowel movements had higher levels of MCP-4 compared to the cases without pain with bowel movements. Among the control participants, we observed significant inverse associations between the presence of dyspareunia and IL-8 and MCP-4, the frequency of dyspareunia and IL-16 and IP-10, and the presence of pain with bowel movements and IL-8.

Interestingly, we did not observe any significant associations in the main analyses between pain symptoms and cytokines or chemokines among the participants with endometriosis. To date, few studies have examined blood-based inflammatory markers in relation to pelvic pain symptoms [30,31,34]. It may be that while the inflammatory milieu differs among endometriosis cases and controls, among those with endometriosis, inflammation is not quantitatively different based on pain symptoms. Further, past studies did not investigate potential effect modification by analgesic use. When stratified by analgesic use at the blood draw, we observed significant associations between cytokines and chemokines and pelvic pain symptoms among the endometriosis cases, suggesting that changes in the inflammatory environment due to analgesic use may alter inflammatory responses in relation to pelvic pain. Specifically, the inverse association between IL-8 and presence of acyclic pelvic pain was only observed among the endometriosis cases who were taking analgesic medications at the blood draw. IL-8 is a pro-inflammatory chemokine that acts as a chemoattractant, particularly for neutrophils [36,37]. Previous research results on IL-8 and pelvic pain symptoms among those with endometriosis have been mixed, with some studies reporting no associations [25,31,38] and a recent study reporting a positive association between IL-8 and dysmenorrhea, post-menstrual pain, and dyspareunia [32]; however, none of these studies considered the effects of analgesic use on the associations between IL-8 and pelvic pain.

Additionally, we observed increased levels of IL-6 among the endometriosis cases with severe compared to no/mild dysmenorrhea among those who were taking analgesics. IL-6 is a pro-inflammatory cytokine with wide-ranging effects on inflammation and the immune response, including B and T cell activation [39]. Previous studies of IL-6 and dysmenorrhea are limited, with one study reporting no association between peritoneal fluid IL-6 levels and presence of dysmenorrhea [32] and another study observing a positive correlation between peritoneal fluid IL-6 levels and dysmenorrhea severity [38]. These discrepancies could be due to differences in how hormonal and analgesic use was handled in these studies, as well as potential differences between the influence of systemic and local cytokines on dysmenorrhea. Further, we observed increased levels of MCP-4, a chemoattractant for monocytes, eosinophils, and basophils [40], among the cases with pain with bowel movements compared to those without among the endometriosis cases who were not taking analgesics. These results suggest that the thoughtful consideration of the potential effects of analgesic use on inflammatory marker levels is needed in pelvic pain research.

While effect modification by analgesic use could explain some of the lack of associations between cytokines and chemokines and pelvic pain among endometriosis cases overall, the lack of associations could also have been related to the fact that only 2% of the endometriosis cases had deep lesions, in which immune cells have been shown to be more enriched compared to superficial lesions [41]. Future research should compare endometriosis patients with deep lesions to those without to better understand the role of cytokines and chemokines in influencing pelvic pain symptoms among endometriosis patients or confirm a true lack of association.

Among the controls, we observed lower IL-8 and MCP-4 levels among those with dyspareunia and those with pain with bowel movements. While a recent study noted a positive association between IL-8 and dyspareunia among infertile patients with endometriosis [32], no direct evidence exists on the relationship between IL-8 or MCP-4 and dyspareunia among individuals without endometriosis. Additionally, IL-16 and IP-10 levels decreased with an increasing dyspareunia frequency among the controls. IL-16 is a pro-inflammatory cytokine that acts as a chemoattractant for CD4+ T lymphocytes, monocytes, and eosinophils [42], while IP-10 has been shown to perform both pro- and anti-inflammatory actions, including chemotaxis, apoptosis, cell growth inhibition, and angiostasis [43]. Few studies have assessed the relationship between IL-16 and pelvic pain, with most studies focusing on patients with endometriosis [44,45,46]. Our endometriosis case-specific observations add to the conflicting results that have been reported for IL-16 levels in the blood and peritoneal fluid of women with endometriosis. One study observed increased levels of IL-16 in the peritoneal fluid of women with endometriosis compared to those without [44], while another small study with 22 endometriosis cases and 22 controls observed no differences in the blood or peritoneal fluid levels of IL-16 between those with endometriosis and those without [45]. Additionally, differences in the IL-16 gene have been associated with differing pain levels among people with endometriosis [46]. These results, in addition to ours, suggest that the relationship between IL-16 and dyspareunia may differ between healthy controls and those with endometriosis, warranting further investigation.

Finally, we observed multiple borderline significant associations between pelvic pain symptoms and the inflammatory biomarkers, which may have been driven by real but small effects that our population was underpowered to detect. The further investigation of these suggestive associations using larger sample sizes would help to increase our understanding of the interplay between pelvic pain and inflammation not only for those with a diagnosed condition, such as endometriosis, but also for women experiencing pelvic pain without a clear diagnosis.

Our study had several strengths, including having a large sample size compared to the previously published studies, with 72% of the participants aged ≤ 25 [47]. Approximately two-thirds of adults with endometriosis report that their symptoms started in adolescence [48,49], meaning that our study population may have been in a more etiologically relevant window for endometriosis development compared to the participants in previous studies, which were mainly conducted among adults many years after their symptoms began. Our study population also predominately presented with rASRM stage I/II disease with superficial peritoneal lesions only, which is the most common endometriosis subphenotype [35]. Additionally, we utilized detailed measures of dysmenorrhea, dyspareunia, acyclic pelvic pain, and pain with bowel movements from the standardized World Endometriosis Research Foundation Endometriosis Phenome and Biobanking Harmonization Project (WERF EPHect) questionnaire to fully investigate the associations between inflammation markers and different types of pelvic pain. Further, our controls were not restricted to those with an indication for surgical evaluation, and thus were less likely to have had a non-endometriosis gynecologic/pelvic condition with a similar symptom presentation that could have had a similar inflammatory milieu profile to that of endometriosis, which would erroneously drive associations towards the null [10]. Finally, the plasma samples utilized in these analyses were collected, processed, and stored following WERF EPHect protocols, and we included blinded quality control samples within the assay batches to assess the assay performance, thereby maximizing the reliability and validity of the inflammation results at each step of the biomarker measurement process [50].

Our study also had limitations. First, some of the inflammation markers measured by the multiplex array had poor reproducibility (high coefficient of variation), particularly TNF-α, suggesting that future analyses are warranted to confirm our results. However, multiplex assays allow for the measurement of multiple markers at the same time, thus efficiently utilizing plasma samples with small volumes and reducing costs. Also, some of the controls within our study may have had undiagnosed endometriosis; however, it is estimated that the community prevalence of undiagnosed endometriosis is <2% [51], and the characteristics of this small proportion of undiagnosed cases would have been diluted by those of the truly endometriosis-free comparison participants. Additionally, although all the analyses were hypothesis-driven based on existing evidence and planned a priori, we performed many statistical tests, meaning that some of our significant associations may have occurred by chance. However, our results stayed largely consistent in the sensitivity analyses. Future analyses are still needed to confirm our results. Finally, these analyses were cross-sectional, meaning that we could not directly elucidate the cause and effect relationship between inflammatory markers and pain symptoms.

Overall, we observed alterations in cytokine and chemokine levels among the controls in relation to dyspareunia and pain with bowel movements but did not observe any significant associations between pelvic pain and inflammation among the endometriosis cases. However, there were significant associations among the endometriosis cases between pelvic pain symptoms and cytokines and chemokines when assessing the relationships separately by whether endometriosis cases were using analgesics at the blood draw, suggesting that analgesic use may modify the associations between pelvic pain and inflammation. The complex relationship between inflammation and pelvic pain, including among those without a diagnosis of endometriosis, warrants further research.

## 4. Materials and Methods

Between 2012 and 2018, the Women’s Health Study: From Adolescence to Adulthood (A2A) enrolled adolescents and adults, oversampling for those surgically diagnosed with endometriosis [52]. Briefly, endometriosis cases (*n* = 787) were enrolled from Brigham and Women’s Hospital (BWH) and Boston Children’s Hospital (BCH) and were eligible if they were (1) of female sex; (2) aged 7–55 years; and (3) had a surgical diagnosis of endometriosis. The controls (*n* = 762) were females aged 7–55 years without a surgical diagnosis of endometriosis, recruited from clinics at BCH and BWH and from the local Boston community through local advertisements, online posts, and word of mouth. Of the 762 controls, 82% were community-based and 18% were clinic-based controls. The study was approved by the BCH Institutional Review Board on behalf of both BCH and BWH. Informed consent was obtained, along with both parental consent and participant assent for participants less than 18 years of age at enrollment.

At enrollment, the participants completed an extensive baseline questionnaire to assess their behavioral and reproductive factors, pain symptoms, quality of life, and medication use, with this questionnaire expanding upon the World Endometriosis Research Foundation (WERF) Endometriosis Phenome and Biobanking Harmonization Project (EPHect) standard clinical questionnaire [53]. The survey data were managed using REDCap electronic data capture tools [54].

### 4.1. Pain Symptom Assessment

Through the baseline questionnaire, detailed information was collected on the presence, severity, and frequency of dysmenorrhea (pain with periods), acyclic/general pelvic pain (pain not associated with menses), and dyspareunia (pain with vaginal sexual intercourse/penetration), as well as pain with bowel movements. For dysmenorrhea, the usual severity was assessed categorically as none, mild (medication was never or rarely needed), moderate (medication was usually needed), or severe (medication and bed rest were needed). The frequency of dysmenorrhea within the past 12 months was assessed as never, occasionally, often, usually, or always. The participants reported whether they had experienced acyclic pelvic pain within the past three months, and an 11-point numeric rating scale (NRS) was used to assess the severity of their acyclic pelvic pain in the past three months, with 0 = no pain and 10 = the worst pain imaginable. Additionally, the frequency of acyclic pelvic pain within the past three months was assessed as being less than monthly, monthly, weekly, or daily. Regarding dyspareunia, participants who were 18 years or older and reported being sexually active indicated if they had experienced dyspareunia in the last 12 months, the severity of their dyspareunia in the last 12 months on the 11-point NRS, and the frequency of their dyspareunia during or in the 24 h after intercourse/penetration in the past 12 months as never, occasionally, often, usually, or always. Finally, participants reported whether they had experienced pain with bowel movements in the past 12 months.

### 4.2. Blood Collection

Blood samples were collected at baseline following the standard protocols of WERF EPHect fluid biospecimen collection with one deviation from the protocol (i.e., blood was centrifuged at 1790× *g* for 10 min) [50]. The participants completed a biospecimen questionnaire at the time of sample collection in which they reported the date of their last menstrual period, the timing of the last food/beverages they had consumed, and recent medication and hormone use. All the blood samples were processed into plasma, serum, and buffy coats and stored at −80 °C.

### 4.3. Cytokine and Chemokine Measurement

**Figure 6:**
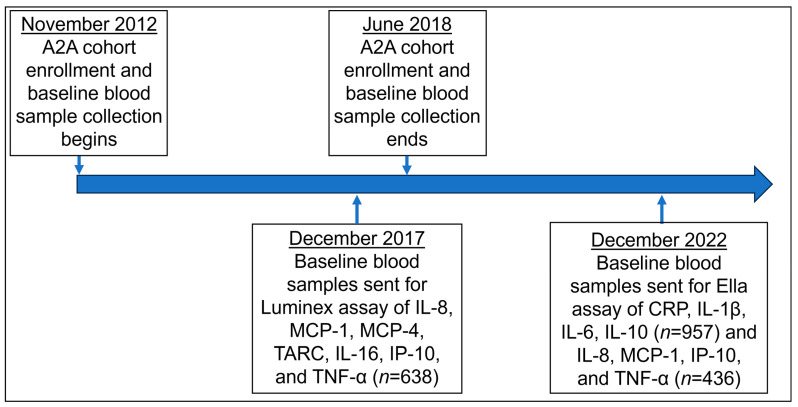
Timeline of A2A cohort enrollment and blood sample collection in relation to assaying of inflammatory markers using Luminex and Ella assays.

Figure 6 shows the timing of the A2A cohort’s enrollment and blood sample collection in relation to when the two assay platforms were used to quantify the inflammatory biomarkers. The plasma samples were sent in two sets at different time points to be assayed, utilizing the Luminex Multiplex platform for the first set and the Ella Immunoassay platform for the second set.

The first set of samples (*n* = 638) was assayed at the laboratory of Dr. Otto Martinez-Maza at the University of California Los Angeles, Los Angeles, CA, USA, in March 2018. This assay utilized two Luminex Multiplex Assay panels (R&D Systems, Minneapolis, MN, USA): a high-sensitivity Luminex panel for human inflammatory cytokines, which included interleukin (IL)-8 and tumor necrosis factor (TNF)-α, and a Luminex panel for human chemokines, which included monocyte chemotactic protein (MCP)-1 and -4, thymus and activation-regulated chemokine (TARC), IL-16, and interferon gamma-induced protein (IP)-10. Briefly, the Luminex xMAP system contains color-coded beads in a 96-well plate to form an antibody–antigen sandwich. A Bio-Plex 200 Luminex array reader (BioRad, Hercules, CA, USA) was used to read the beads within each well to determine the analyte and signal magnitude using BioPlex Manager (v 4.1.1) software. For each analyte, the lower limit of detection was set as the lowest value that could be calculated using the standard curve function in BioPlex Manager software.

The second set of samples was assayed at the laboratory of Dr. Towia Libermann at Beth Israel Deaconess Medical Center, Boston, MA, USA, in February 2022 using three Ella Immunoassays (R&D Systems, Minneapolis, MN, USA) utilizing the Ella Automated Immunoassay System. Briefly, Ella Immunoassays use an automated microfluidic enzyme-linked immunosorbent assay system to detect biomarker concentrations. In the Ella Immunoassay, we measured the levels of IL-8, TNF-α, MCP-1, and IP-10 in 436 samples and C-reactive protein (CRP), IL-1β, IL-6, and IL-10 in 957 samples.

### 4.4. Assay Quality Control

Approximately 2–6 blinded quality control (QC) plasma samples were distributed randomly within each batch. In the Luminex assay, the coefficient of variation (CV) for the blinded QC samples was <10% for MCP-1 and IP-10 and between 10 and 15% for IL-8, IL-16, TNF-α, and TARC. In the Ella Immunoassay, the CV for the blinded QC samples was <10% for MCP-1, IL-6 and IP-10, 10–15% for CRP, IL-8, IL-10, and TNF-α, and 41% for IL-1β.

### 4.5. Recalibration of Inflammatory Marker Values

As four markers (IL-8, TNF-α, MCP-1, and IP-10) were assayed across two different platforms, we included drift samples within the Ella Immunoassay to recalibrate the biomarker concentrations across the two platforms. Briefly, 117 participants with plasma samples analyzed using the Luminex assay and encompassing a wide range of biomarker concentrations were included in the Ella Immunoassay. Before recalibrating the values, we assessed the correlation between the Luminex and Ella Immunoassay values for each of the four markers. As the Pearson correlation was moderate to very strong for IL-8 (r = 0.58; *p* < 0.001), MCP-1 (r = 0.75; *p* < 0.001), and IP-10 (r = 0.82; *p* < 0.001), we recalibrated the assay values for these markers. To recalibrate the first set of inflammatory values measured in the Luminex assays to equivalent Ella Immunoassay values, we used linear regression and regressed the original Luminex assay values on the new Ella Immunoassay values to obtain the corresponding slope and intercept for each inflammatory biomarker [55]. The slope and intercept were then used to predict the recalibrated Ella Immunoassay values for all the participants analyzed in the Luminex assay for IL-8, MCP-1, and IP-10. As the distributions for TNF-α differed across the two platforms (r = 0.30), we have presented the results separately by platform for TNF-α in the secondary analyses.

### 4.6. Covariates

Descriptive characteristics were collected in the baseline questionnaire and the biospecimen form and included participants’ age (continuous), race (Black, White, other/unknown race), smoking history (never, former, current), age at menarche (continuous), menstrual cycle phase at the time of the blood draw among those not using hormonal medications (follicular, peri-ovulatory, luteal), hormonal medication use within 30 days of the blood draw (yes, no), and analgesic pain medication use within 48 h of the blood draw (yes, no). The participants reported the frequency of their menstrual periods in the past 12 months, including whether they had had a period within the past three months. The body mass index (BMI) was calculated as kg/m^2^ based on participants’ self-reported weight and height. For women aged >20 years, their BMI was categorized according to the World Health Organization Criteria as underweight (BMI < 18.5 kg/m^2^), a normal weight (18.5–24.9 kg/m^2^), overweight (25–29.9 kg/m^2^), or obese (>30 kg/m^2^). For those <20 years old, their age- and sex-specific BMI Z-score was calculated, and participants were categorized as underweight (Z-score < −2), a normal weight (Z-score > −2 to <1), overweight (Z-score 1–2), or obese (Z-score > 2). Information on participants’ rASRM stage and endometriosis subphenotype at the time of the surgery they had closest to the blood draw were extracted from the WERF EPHect surgical form for endometriosis cases [56] and medical records, as well as their reported age at first endometriosis symptoms, the number of physicians seen before being diagnosed, and the time between their first symptoms and surgical diagnosis, extracted from the baseline questionnaire. 

### 4.7. Analytic Study Population

Table 3 displays the final analytic sample size for each inflammation biomarker after exclusion due to non-detectable assay levels, outliers, a high intra-batch CV of >30%, the timing of baseline questionnaire completion (>90 days before/after the blood draw), and the use of immune-modulating drugs at the time of the blood draw. The exclusions for TNF-α are presented in Table S11. Regarding the dysmenorrhea analyses, we additionally restricted these to participants who reported having had menstrual periods in the past three months. Analyses of acyclic pelvic pain severity and frequency were restricted to participants who reported having had acyclic pelvic pain in the past 3 months. Finally, analyses of dyspareunia severity and frequency were restricted to participants who reported having experienced dyspareunia in the past 12 months. The sample sizes for each analysis described below can be found in the supplemental tables.

### 4.8. Statistical Analysis

We used the generalized extreme Studentized deviate many-outlier detection approach to identify statistical outliers for each inflammatory marker assayed [41]. After these exclusions, the levels of TNF-α, which had a higher CV, were adjusted to have a comparable distribution to an average batch according to the methods described by Rosner and colleagues [57]. The biomarker levels were natural log-transformed to improve normality. Missing values for covariates were imputed to the most common category for BMI (<1% missing; imputed to a normal BMI) and hormone use within 30 days of the blood draw (1% missing; imputed to yes). A missing indicator was included for analgesic pain medication use within the 48 h prior to the blood draw as 5% of participants were missing this covariate. We compared the inflammatory marker levels between endometriosis cases and controls utilizing linear regression and adjusting for participants’ age (continuous), BMI (underweight, normal weight, overweight, obese), hormone use within the 30 days prior to the blood draw (yes, no), and analgesic pain medication use within the 48 h prior to the blood draw (yes, no, missing). We additionally adjusted for severity of dysmenorrhea (none/mild, moderate, severe) and the presence of acyclic pelvic pain (yes, no) to account for the influence of pain symptoms on the relationship between the endometriosis case–control status and each of the inflammatory marker levels.

In the main analyses, we used linear regression for participants with and without endometriosis separately to assess the association between each pain symptom (independent variable) and each cytokine or chemokine (IL-6, -8, -10, and -16, MCP-1 and -4, TARC, and IP-10; dependent variable) and calculated the geometric means and 95% confidence intervals (CIs) for each cytokine and chemokine, adjusting for participants’ age (continuous) and BMI (underweight, normal weight, overweight, obese) in Model 1 and additionally adjusting for hormone use within the 30 days prior to the blood draw (yes, no) and analgesic pain medication use within the 48 h prior to the blood draw (yes, no, missing) in Model 2. Trend tests were performed by modeling the categorical exposure variables (i.e., dysmenorrhea severity and frequency, acyclic pelvic pain severity, and dyspareunia severity and frequency) as ordinal variables adjusted for the same covariates. To investigate whether the associations between the pain exposure variables and inflammatory markers differed between the cases and controls, we conducted linear regression models including an interaction term for the pain exposure variable and case–control status and calculated the *p*-value for the interaction using the two-sided Wald statistic.

In the secondary analyses (presented in the supplemental tables only), we assessed the associations between the pelvic pain characteristics and TNF-α and IL-1β levels. For TNF-α, we utilized the same methods as described above with the exception that we assessed the associations separately for TNF-α levels determined using the Luminex and Ella platforms. As the CV for IL-1β was high (41%), we categorized IL-1β levels as being below the limit of detection, between the limit of detection and the median, or above the median. We utilized polytomous logistic regression to analyze the association between pain symptoms and the three categories of IL-1β levels (below the limit of detection, between the limit of detection and the median, above the median), adjusting for participants’ age (continuous) and BMI in Model 1 and additionally adjusting for hormone use within the 30 days prior to the blood draw (yes, no) and analgesic pain medication use within the 48 h prior to the blood draw (yes, no, missing) in Model 2. To investigate whether the associations between the pain exposure variables and IL-1β differed between the cases and controls, we conducted polytomous logistic regression models including an interaction term for the pain exposure variable and case–control status and calculated the *p*-value for the interaction using the two-sided Wald statistic.

To assess effect modification by analgesic pain medication use within the 48 h prior to blood collection, we conducted analyses stratified by analgesic pain medication use separately for the cases and controls. As this stratification reduced the sample size for certain categories of the pain variables, we conducted the stratified analyses on the main pain variables: dysmenorrhea severity, presence of acyclic pelvic pain, presence of dyspareunia, and pain with bowel movements. To investigate whether the associations between the pain exposure variables and inflammatory markers differed by the analgesic pain medication use, we included an interaction term for the pain exposure variable and analgesic pain medication use and calculated the *p*-value for the interaction using the two-sided Wald statistic.

We performed a number of sensitivity analyses. We excluded participants who reported using steroid medications within 48 h of blood collection to remove the potential effects of steroid hormones on the inflammatory biomarker levels. Additionally, we conducted analyses excluding participants with extrapolated values for TARC (7% of samples), IL-10 (6% of samples), IL-6 (3% of samples), and IL-8 (2% of samples) to assess the effect of these extrapolated values on the results. Finally, we excluded endometriosis cases who had an endometriosis-related surgery before they completed their baseline questionnaire and/or their baseline blood draw to remove the effects that surgery may have had on their inflammation levels and pain symptoms. This exclusion substantially reduced the sample size for certain categories of the pain variables; therefore, we conducted sensitivity analyses excluding those who underwent an endometriosis-related surgery before completing their questionnaire or blood draw and utilizing the main pain variables: dysmenorrhea severity, presence of acyclic pelvic pain, presence of dyspareunia, and pain with bowel movements. The results with this exclusion were similar to the main results and are presented in Tables S12 and S13.

All the statistical analyses were performed using SAS version 9.4 (SAS Institute Inc., Cary, NC, USA), and all the *p*-values are two-sided.

## Figures and Tables

**Table 1:** Descriptive characteristics of A2A participants with at least one inflammatory biomarker identified in plasma samples (*n* = 894).

Characteristics	Endometriosis (*n* = 389)	No Endometriosis (*n* = 505)
Age at blood draw, years		
Median (IQR)	17 (16–21)	24 (22–29)
Race, *n* (%)		
White	353 (91%)	366 (72%)
Black	9 (2%)	33 (7%)
Other/unknown ^1^	27 (7%)	106 (21%)
Body mass index (kg/m^2^), *n* (%) ^2,3^		
Underweight	4 (1%)	18 (4%)
Normal weight	236 (61%)	328 (65%)
Overweight	110 (28%)	106 (21%)
Obese	39 (10%)	52 (10%)
Smoking status, *n* (%) ^2^		
Never	346 (95%)	456 (92%)
Ever	20 (5%)	37 (8%)
Age at menarche, years		
Median (IQR)	12 (11–13)	12 (11–13)
Menstrual cycles in the last 3 months, *n* (%) ^4^		
No	135 (35%)	78 (15%)
Yes	250 (65%)	427 (85%)
Menstrual cycle phase at blood draw, *n* (%) ^2,5^		
Follicular	17 (49%)	71 (42%)
Peri-ovulation	6 (17%)	24 (14%)
Luteal	12 (34%)	73 (43%)
Hormonal medication use in the last 30 days prior to the blood draw, *n* (%) ^2^		
Not using hormones	48 (12%)	219 (44%)
Using hormones	340 (88%)	278 (56%)
Pain medication use in the last 48 h prior to the blood draw, *n* (%) ^2^		
No	269 (77%)	401 (81%)
Yes	81 (23%)	96 (19%)
Steroid medication use in the last 48 h prior to the blood draw, *n* (%) ^2,6^		
No	329 (94%)	468 (94%)
Yes	21 (6%)	29 (6%)
rASRM stage, *n* (%) ^2^		
Stage I/II	360 (95%)	--
Stage III/IV	18 (5%)	--
Endometriosis subtype, *n* (%) ^2^		
Superficial peritoneal lesions only	364 (95%)	--
Endometrioma	8 (2%)	--
Deep infiltrating	6 (2%)	--
Endometrioma and Deep	1 (0%)	--
Age at first endometriosis symptoms, years		
Median (IQR)	13 (12–15)	--
Time between first symptoms and surgical diagnosis, years		
Median (IQR)	2 (1–5)	--
Number of doctors seen for symptoms before diagnosis		
Median (IQR)	2 (1–4)	--

^1^ Participants in the other/unknown category were Asian (1 case, 67 controls), Native American (1 case), Hawaiian/a Pacific Islander (1 control), more than one race (12 cases, 25 controls), another race (12 cases, 10 controls), or an unknown race (1 case, 3 controls). ^2^ Numbers for the different categories do not all add up to 389 endometriosis cases and 505 controls due to missing values (BMI: controls = 1; smoking: cases = 23, controls = 12; menstrual cycle phase: cases = 2, controls = 10; menstrual cycles in the last 3 months: cases = 4; hormone use: cases = 1, controls = 8; analgesic pain medication use: cases = 39, controls = 8; steroid medication use: cases = 39, controls = 8; endometriosis rASRM stage: cases = 11; endometriosis subtype: cases = 10. ^3^ Women aged ≥ 20 years were classified as underweight (BMI < 18.5 kg/m^2^), a normal weight (BMI of 18.5–24.9 kg/m^2^), overweight (BMI of 25–29.9 kg/m^2^), or obese (BMI ≥ 30 kg/m^2^) according to the World Health Organization criteria; for those <20 years old, their age- and gender-specific BMI Z-score was calculated, and participants were categorized as underweight (Z-score ≤ −2), a normal weight (Z-score > −2 to <1), overweight (Z-score of 1–2), or obese (Z-score > 2). ^4^ Due to the phrasing in the questionnaire, participants who self-reported having periods could have been on cyclic hormone therapy or experienced bleeding despite being on continuous hormones. ^5^ Among the participants not using hormones with self-reported menstrual periods in the past 3 months, excluding those who reported cycle lengths < 21 days or >35 days. ^6^ Steroid medications included corticosteroids, glucocorticosteroids, mineralocorticoids, hydrocortisone, and other unspecified steroids.

**Table 2:** Cytokine and chemokine levels among A2A participants with and without endometriosis ^1^.

Inflammation Marker	Endometriosis		No Endometriosis		
	N	Geometric Mean (95% CI)	N	Geometric Mean (95% CI)	*p*-Value ^2^
CRP	368	1.54 (1.34, 1.78)	486	1.26 (1.11, 1.42)	0.05
IL-6	384	1.59 (1.49, 1.70)	504	1.63 (1.55, 1.73)	0.55
IL-8	389	5.10 (4.96, 5.26)	505	4.87 (4.75, 5.00)	0.03
IL-10	382	1.82 (1.75, 1.90)	501	1.98 (1.91, 2.05)	0.005
MCP-1	389	173.7 (168.5, 179.0)	504	165.3 (161.1, 169.7)	0.03
MCP-4	307	77.3 (71.7, 83.4)	295	70.1 (64.9, 75.7)	0.10
TARC	299	230.2 (210.2, 252.2)	288	231.0 (210.5, 253.5)	0.96
IL-16	303	127.9 (122.0, 134.1)	294	122.7 (117.0, 128.98)	0.27
IP-10	383	119.3 (113.0, 125.8)	505	109.9 (105.0, 115.1)	0.04

^1^ Geometric means adjusted for age (continuous), BMI (under-/normal weight, overweight, obese), hormone use within 30 days prior to blood draw (yes, no), and pain medication use within 48 h prior to blood draw (yes, no, missing). ^2^ *p*-value for difference in geometric mean levels of each inflammation marker by case–control status.

**Table 3:** Reasons for exclusion and final analytic sample size for each inflammatory marker in the baseline blood samples from the A2A cohort.

	Luminex Assay Only			Ella Immunoassay and Luminex Recalibration ^1^			Ella Immunoassay Only			
	MCP-4	TARC	IL-16	IL-8	MPC-1	IP-10	CRP	IL-1β	IL-6	IL-10
Inflammation marker	ca/co	ca/co	ca/co	ca/co	ca/co	ca/co	ca/co	ca/co	ca/co	ca/co
Blood samples sent to laboratory (cases/controls)	335/303	335/303	335/303	431/526	431/526	431/526	431/526	431/526	431/526	431/526
Exclusions										
Non-detectable levels	--	8/7 *	--	*	--	*	--	**	*	*
Outliers ^2^	--	1/0	4/1	--	0/1	6/0	--	--	5/1	8/5
Intra-assay batch CV > 30%	--	--	--	--	--	--	23/19	--	--	--
Baseline questionnaire completed 90 days before/after blood draw	22/4	21/4	22/4	33/10	33/10	33/10	31/10	33/10	33/10	33/9
Using immune-modulating drugs at time of blood draw	6/4	6/4	6/4	9/11	9/11	9/11	9/11	9/11	9/11	8/11
Final analytic sample	307/295	299/288	303/294	389/505	389/504	383/505	368/486	389/505	384/504	382/501

* Samples below the limit of detection were extrapolated for some of the inflammatory markers: TARC—25 cases/21 controls; IL-8—10 cases/4 controls; IP-10—1 case; IL-6—15 cases/12 controls; IL-10—29 cases/27 controls. These participants were included in the main analyses but removed for the sensitivity analyses. ** For IL-1β, 242 endometriosis cases and 237 controls had values below the lower limit of detection, which were incorporated into the bottom category of the categorical variable created for the IL-1β levels. ^1^ For IL-8, MCP-1, and IP -10, 335 cases and 303 controls were assayed using the Luminex platform, while 431 cases and 526 controls were assayed using the Ella Immunoassay. Samples were recalibrated between the two assays, utilizing 117 samples on which both assays were run. In the final analytic sample set, the Luminex assay was run on 279 cases and 212 controls for IL-8, 279 cases and 212 controls for MCP-1, and 274 cases and 212 controls for IP-10, with the Ella Immunoassay run on those remaining. ^2^ Outliers were detected utilizing the generalized extreme Studentized deviate many-outlier detection approach.

## Data Availability

The data are not publicly available due to containing information that could compromise the research participants’ privacy and consent. However, experienced scientists who would like to make an enquiry regarding the use of the data from this study to address specific hypotheses or replicate the analyses performed in this study may submit an application and research proposal. Data requests must be reviewed and approved by the BWH Institutional Review Board (https://www.brighamandwomens.org/research/research-administration URL (accessed on 30 May 2025)). All inquiries should be directed to the A2A senior investigator and Boston Center for Endometriosis Scientific Director, Dr. Stacey Missmer (smissmer@hsph.harvard.edu). Data sharing will require a fully executed Data Usage Agreement.

## Supplementary Materials

The following supporting information can be downloaded at https://www.mdpi.com/article/10.3390/ijms26115377/s1.

## Abbreviations

The following abbreviations are used in this manuscript:

A2A	Adolescence to Adulthood cohort
IL	Interleukin
TNF	Tumor necrosis factor
CRP	C-reactive protein
MCP	Monocyte chemotactic protein
TARC	Thymus and activation-regulated chemokine
IP	Interferon gamma-induced protein
GM	Geometric mean
CI	Confidence intervals
rASRM	Revised American Society for Reproductive Medicine
IQR	Interquartile range
BMI	Body mass index
OR	Odds ratio
BWH	Brigham and Women’s Hospital
BCH	Boston Children’s Hospital
WERF	World Endometriosis Research Foundation
EPHect	Endometriosis Phenome and Biobanking Harmonization Project
NRS	Numeric rating scale
QC	Quality control
CV	Coefficient of variation
